# Enhanced Mechanical Properties and Oxidation Resistance of Zirconium Diboride Ceramics via Grain‐Refining and Dislocation Regulation

**DOI:** 10.1002/advs.202104532

**Published:** 2022-01-02

**Authors:** Haiyue Xu, Wei Ji, Weiming Guo, Yulin Li, Ji Zou, Weimin Wang, Zhengyi Fu

**Affiliations:** ^1^ State Key Laboratory of Advanced Technology for Materials Synthesis and Processing Wuhan University of Technology Wuhan 430070 China; ^2^ School of Electromechanical Engineering Guangdong University of Technology Guangzhou 510006 China

**Keywords:** dislocation multiplication, grain‐refining, oxidation resistance, plastic deformation, ultra‐high pressure sintering

## Abstract

Zirconium diboride (ZrB_2_) is considered as one of the most promising ultra‐high temperature materials for the applications in extreme environments. However, the difficulty in fabrication of ZrB_2_ limits its industrial applications. In this study, fully dense and grain‐refined ZrB_2_ is prepared under ultra‐high pressure of 15 GPa at low temperature of 1450 °C. The as‐prepared ZrB_2_ exhibits excellent mechanical and oxidation‐resistant properties. Compared with raw powder, the grain size decreases 56%. Compared with high‐temperature sintered control specimen beyond 2000 °C, the hardness and fracture toughness increase about 46% and 69%, respectively, the dislocation density increase 3 orders of magnitude, while the grain size considerably decrease 96%. According to work hardening, Hall–Petch and Taylor dislocation hardening effects, the refined grains, substructures, and high dislocation density caused by plastic deformation during sintering can enhance the mechanical properties. The unique structure contributes to a threshold oxidation temperature increase of ≈250 °C relative to the high‐temperature sintered ZrB_2_, achieving one of the highest values (1100 °C) among the reported monolithic ultra‐high temperature ceramics. A developed densification mechanism of dislocation multiplication with grain refining is proposed and proved to dominate the sintering, which is responsible for simultaneous improvements in mechanical and oxidation‐resistant properties.

## Graphical abstract

Grainsize‐decreased and full‐density ultra‐high‐temperature materials are prepared under extreme condition of ultra‐high pressure. The specimen exhibits excellent mechanical properties (hardness increases 46%, fracture toughness increases 69%) and oxidation resistance (oxidation temperature rises 250 °C). Dislocation multiplication with grain‐refining is proved to dominate the fabrication process, which is responsible for the simultaneous improvements in mechanical and oxidation‐resistant properties.

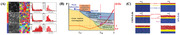

## 1 Introduction

Boride ceramics are an emerging materials for the applications at ultra‐high temperatures.^[^ ^1^ ^]^ They exhibited high hardness, high stiffness, high strength, and well oxidation resistance at elevated temperature and possessed high electrical and thermal conductivities. The combination of metal‐like and ceramic‐like allows them suitable for thermal protection system of hypersonic flight, rocket propulsion, and motors.^[^ ^2^, ^3^ ^]^ However, due to the intrinsically strong covalent bonds and high melting point (>3000 °C),^[^ ^4^ ^]^ it is difficult to obtain fully dense monolithic boride ceramics with fine grains mainly because of their low diffusion coefficient.^[^ ^5^, ^6^ ^]^

Pressureless sintering of ZrB_2_, a typical boride ceramic, is still a significant challenge. The densification could be improved by prolonging holding time or supplying kinds of sintering additives.^[^ ^6^, ^7^ ^]^ Chamberlain reported that the relative density could be enhanced from 73% to 98% at 2150 °C with the holding time from 3 to 9 h.^[^ ^6^ ^]^ Additionally, the porosity of ZrB_2_ densified by pressureless sintering with B_4_C as additive (5 vol%) at 2150 °C was reduced from 30% to 5%.^[^ ^7^ ^]^ Pressure‐assisted sintering methods such as hot pressing (HP) and spark plasma sintering (SPS) were encouraged and used in industry to prepare full density ZrB_2_ with simple shapes.^[^ ^8^, ^9^, ^10^, ^11^, ^12^, ^13^ ^]^ With HP technology, ZrB_2_ ceramics with the relative density of 97% were obtained after sintering at 2200 °C for 2 h under 50 MPa.^[^ ^8^ ^]^ SPS technology could facilitate densification and improve mechanical properties of ZrB_2_ with the assistance of electrical field.^[^ ^10^, ^11^, ^12^, ^13^ ^]^ Chakraborty reported that a high relative density of 98.65% was achieved with pulsed direct current by controlling 50 ms pulse on and 5 ms pulse off mode.^[^ ^10^ ^]^ Densified ZrB_2_ ceramics with the relative density of 80–97% were obtained by SPS at 1800 to 1950 °C.^[^ ^11^ ^]^ However, the residual pores and large grains formed in the final stage still may decrease the mechanical and oxidation‐resistant properties of the obtained ZrB_2_ ceramics.

To date, high pressure sintering has been regarded as an efficient method to fabricate ceramics.^[^ ^14^, ^15^, ^16^, ^17^, ^18^, ^19^ ^]^ Ji's group prepared fully dense micro‐sized B_4_C without grain growth under 80 MPa at 1700 °C.^[^ ^14^ ^]^ Xu et al., fabricated 290 nm alumina under 200 MPa at 1000 °C. In these researches, plastic deformation was proposed and proved as the dominant mechanism in high‐pressure sintering.^[^ ^15^ ^]^ Gu and co‐workers sintered micro‐sized TaC ceramics with a high melting point under 250 MPa at 1850 °C. But the grain size severely grew up above 5 times.^[^ ^16^ ^]^

Tian et al., synthesized ultra‐hard nanotwinned cubic boron nitride (c‐BN) from onion‐like BN (oBN) nanosphere under the conditions of 1800 °C and 15 GPa.^[^ ^17^ ^]^ The same research group then fabricated nanotwinned diamond with hardness of 200 GPa from onion carbon particles at 2000 °C under 20 GPa.^[^ ^18^ ^]^ The unique microstructures make them possess high fracture toughness and thermal stability.^[^ ^20^ ^]^ However, for ceramic materials, the densification mechanism during ultra‐high pressure sintering (UHPS), and the effects of the extremely high pressure on grain size, dislocation density, and related properties in practical application environment have not been well developed. It is expected that UHPS strategy could fully densify ultra‐high temperature ceramics with fine grains and promising properties associated with new sintering mechanisms.

In this work, fully dense ZrB_2_ monolithic ceramics with decreased grain size were prepared by using the UHPS method. The densification, microstructure evolution, mechanical properties, and oxidation resistance of the as‐prepared ceramics were well studied. The relationship among hardness, grain size, and dislocation density were explored in this study. The sintering mechanisms under ultra‐high pressure and their influences on properties were also discussed.

## 2 Results and Discussion

### 2.1 Densification and Microstructural Evolution under Ultra‐High Pressure

**Figure 1:**
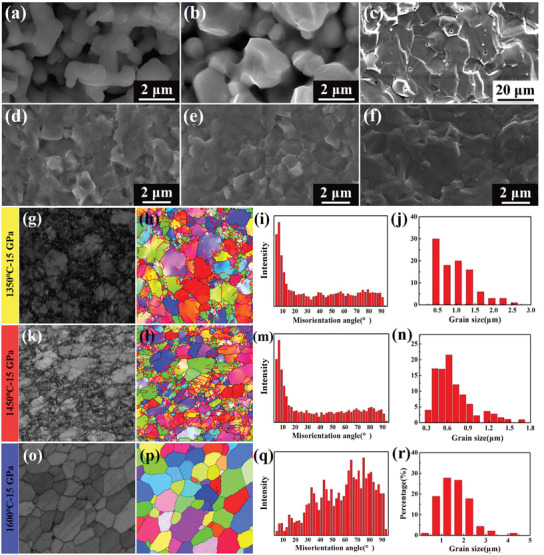
SEM images of a) raw powder. The fracture surfaces of SPS‐ed ZrB_2_ under the conditions of b) 1800 °C and 50 Mpa and c) 2000 °C and 200 MPa. The fracture surfaces of UHPS‐ed ZrB_2_ under 15 GPa at d)1350, e)1450, and f)1600 °C. EBSD results including the band contrast figures, inverse pole figures, misorientation angle distributions, and grain size distributions of ZrB_2_ ceramics sintered under 15 GPa at g–j) 1350, k–n)1450, o–r)1600 °C. In (h,l,p), the black lines represent high‐angle grain boundaries (>15°) while the red lines represent small‐angle grain boundaries (≤15°).

**Figure** **1**a–c illustrates the SEM images of raw starting powder and the fracture surfaces of the control ZrB_2_ specimen prepared by SPS.^[^ ^21^ ^]^ The pressure of traditional pressure‐assisted sintering is limited below 50 MPa because of the strength of graphite sintering mould. The applied load can be increased to 200 MPa through special mould design. Since ZrB_2_ possesses excellent electrical conductivity, the current is suggested to produce plasma between particles during SPS process, which could facilitate sintering.^[^ ^20^, ^22^, ^23^ ^]^ However, there is only limited densification for the ZrB_2_ sintered at 1800 °C under 50 MPa, as shown in Figure 1b. The relative density only achieved 88% with obvious grain growth. When the sintering parameters were raised to 2000 °C and 200 MPa, the relative density was increased to 99%, while the grain size significantly increased 12 times and reached 19 µm. Dramatic grain growth degrades the mechanical properties. The fracture surface (Figure 1c) shows that many isolated pores were trapped within grains because the migration of grain boundaries at high temperatures was faster than pores.^[^ ^24^ ^]^ The intragranular pores were the main reason to prevent ZrB_2_ ceramics from obtaining fully dense structures. Grain growth was mainly controlled by grain boundary migration, which was a thermal activated process mainly depending on temperature. Holm et al.^[^ ^25^ ^]^ suggested that when temperature was below the onset temperature (*T*_sg_), grain boundary migrated slowly and the grains scarcely grew. When temperature was above *T*_sg_, grain boundary migrated rapidly and grain size increased obviously. The grain coarsening of SPS‐ed specimen in the present work could be probably associated with the rapid grain boundary immigration beyond *T*_sg_ of ZrB_2_ ceramics.

The fracture morphologies of ZrB_2_ samples sintered under 15 GPa at different temperatures (1350, 1450, and 1600 °C) are shown in Figure 1d–f. The microstructures were fine, uniform, and basically free of porosity, indicating that the UHPS method could decrease the sintering temperature of as much as 600 °C than that by SPS method.

Figure 1g–r shows the electron backscatter diffraction analysis probe (EBSD) characterizations of ZrB_2_ ceramics sintered at 1350, 1450, and 1600 °C. When the sintering temperature was 1350 °C, the grain size was measured to be 1.1 µm, finer than the raw powder. A large proportion of small‐angle grain boundaries were observed along the high‐angle grain boundaries or triple‐junctions. The severe plastic deformation under ultra‐high pressure promoted the accumulation of line defects, which eventually transformed into small‐angle grain boundaries.^[^ ^26^ ^]^

When the sintering temperature reached to 1450 °C, most grains were broken to form subgrains, thus leading to high density of low angle grain boundaries, which distributed uniformly in the sample, not only in the grains, but also along original high angle grain boundaries.^[^ ^27^ ^]^ The grain size further decreased from 1.1 to 0.7 µm at 1450 °C, reduced 56% from the raw powder. The increasing sintering temperature promoted the formation and content of new small‐angle grain boundaries and subgrains whereas low‐temperature sintering suppressed grain boundary immigration by reducing atom diffusion and finally refined grains.

When the temperature increased to 1600 °C, the average grain size of ZrB_2_ increased to 1.6 µm, which has returned to the initial powder size again. The increasing temperature could accelerate the diffusion rate of atoms, thus causing the annihilation of dislocations and substructures. The subgrains were transformed into equiaxed grains. The large fraction of high‐angle grain boundaries was developed from low angle grain boundaries. The characterization results of the samples sintered at 1450 and 1600 °C indicated that the internal stress and dislocations were released to activate the grain growth mechanism.^[^ ^28^ ^]^

UHPS technology generally showed two primary advantages over traditional high‐temperature sintering method. First, it could greatly reduce the sintering temperature for achieving fully dense while simultaneously restrain rapid grain boundary migration, preventing the product of intragranular pores and suppressing grain growth. Second, the ultra‐high pressure could also efficiently create new grain boundaries and subgrains, thus leading to grain refinement. This unique microstructure revolution was probably beneficial to mechanical properties and other application properties. Therefore, the combination of improving sintering pressure and reducing sintering temperature is one of the most efficient ways to obtain fully dense nano‐sized ultra‐high temperature ceramics without intragranular pores with excellent properties.

Powder sintering under ultra‐high pressure causes plastic yield and the densification process is dominated by plastic deformation mechanism. According to Coble's tetrakaidecahedron geometrical model, the relationship between average contacting area and external pressure is expressed as:^[^ ^29^, ^30^ ^]^

(1) $$\;A_{r}=\frac{4\pi R^{2}P_{a}}{ZP_{y}}\;$$

where *A*_r_ is the real contacting area of each facet; *P*_a_ is the applied load; *P*_y_ is the fully plastic yield stress (the same as hardness); *R* is the mean grain radius; *Z* is co‐ordination number, 12.

In the situation of ceramics with plastic contacts, besides the compressive stress, the rough surface could also generate friction.^[^ ^31^ ^]^ The applied load could be divided into normal pressure and shear stress caused by friction. In the sintering densification model, the neck area could be regarded as a flat area, so the apparent contact area of per facet is expressed as:^[^ ^32^ ^]^

(2) $$\;A_{a}=\frac{A_{r}}{{\left[1\;+\;\alpha \mu \right]}^{\frac{1}{2}}}\;$$

where *A*_a_ is the apparent contacting area of each facet considering friction condition; *α* is a constant and set as 9; *μ* is the sliding friction coefficient.^[^ ^32^ ^]^

Under the condition that surface diffusion is negligible, the relationship between the final porosity and the apparent neck areas is given by:^[^ ^33^ ^]^

(3) $$\;\frac{\theta_{0}\;-\;\theta }{12\theta_{0}}=\frac{A_{a}}{4\pi R^{2}}\;$$

where *θ*_0_ is the initial porosity and set as 0.36 in this model, *θ* is the final porosity. On the basis of the previous studies, the values of the hardness *H* of ZrB_2_ ceramics sintered at 1350 and 1450 °C are respectively set as 5.5 and 5 GPa, respectively.^[^ ^27^, ^34^ ^]^ The sliding friction coefficient is considered as 0.95.

Based on this densification model, the relative density by plastic deformation at 1350 °C under high temperature could achieve 96.6%. When the temperature was increased to 1450 °C, the calculated relative density reached as high as 98.9%. Since there is almost no grain growth at 1450 °C, the grain boundary immigration by atom diffusion is very limited. During the plastic deformation process, the grains could undergo work‐hardening to coordinate the constant deformation.

This unique microstructure evolution of ZrB_2_ ceramics under ultra‐high pressure was similar to the dynamic continuous recrystallization model for metals, which could be used to process nanomaterial and strengthen the hardness.^[^ ^28^ ^]^ However, because of the lack of slip systems, it is difficult for ceramics to enhance mechanical properties by decreasing the grain size, even under severe plastic deformation. Unprecedently, here the grain size of ZrB_2_ ceramics decreased more than half through UHPS technology.

Among sintering parameters, we need to find the dynamic recrystallization threshold temperature, at which atoms diffusion occurs obviously.^[^ ^28^ ^]^ The ceramics with a high dislocation density, unique microstructure, and excellent mechanical properties could be obtained through sintering at the temperature lower than the threshold point required for initiating dynamic recrystallization. The UHPS technology provides a pathway for low temperature densification, grain size refinement, and mechanical properties improvement.

### 2.2 Sintering Mechanisms under Ultra‐High Pressure

**Figure 2:**
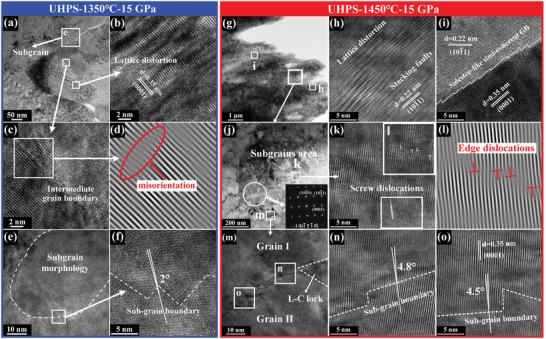
The detailed microstructures of ZrB_2_ sintered at 1350 °C under 15 GPa: a) TEM bright‐field image. b,c,e) HRTEM images of the selected areas marked in (a). d) The inverse Fourier transform (IFFT) pattern of the selected area in (c). f) HRTEM image information of the selected area marked in (e). ZrB_2_ sintered at 1450 °C under 15 GPa: g) TEM bright‐field image, h,i) HRTEM images of the areas (h) and (i) in (g), j) bright‐field image of the selected area in (g), with an inserted SAED pattern reflecting the crystal information in the circular area, k) HRTEM image of the corresponding location in (j), l) the IFFT image of the corresponding area in (k), m) HRTEM image of square area in (j) and n,o) HRTEM images of areas (n) and (o) in (m).

The crystallite breaking effect was not observed obviously for ZrB_2_ sintered under 15 GPa at 1350 °C (**Figure** **2**). The sub‐grain boundary profile of ZrB_2_ densified by severe plastic deformation mechanism was curved and rough. Subgrains were found near high‐angle grain boundaries. The crystals shown in Figure 2b include many dislocation lines, which probably present the initial stage. However, the microstructure observation here clearly shows the intermediate production process of subgrains with low angle grain boundaries. The lattice distortion shown in Figure 2c marks the occurrence of severe plastic deformation of grains, which could be considered as the incubation stage for the formation of sub‐grain boundaries. The angle of sub‐grain along the boundaries was very small (≈2°), which exhibits the formed low angle grain boundary (Figure 2f).

The detailed microstructure in Figure 2g–o depicts that when the temperature increased to 1450 °C, much more severe plastic deformations such as high‐density nano‐sized stacking faults and lattice distortions occurred. The plastic deformation of grain boundary (Figure 2i) showed rough step‐like grain boundary profile. The sub‐grains with small angle misorientation were formed in the ZrB_2_ ceramics, as indicated by the selected area electron diffraction (SAED) pattern obtained from the circular region (Figure 2j). The multiple sets of spots were an indication of nanocrystalline sub‐grains with small misorientation.^[^ ^26^ ^]^ Figure 2k displays a large quantity of screw and edge dislocations formed in the lattice. The accumulation of dislocation lines is the main formation mechanism of subgrains and small‐angle boundaries. The orientation deviation angles between the two sides of small‐angle grain boundary were about 4.5° and 4.8°, respectively in the HRTEM images in Figure 2m–o. Interactions among the massive dislocations resulted in the formation of Lomer–Cottrell locks (Figure 2m), which enhanced the ability of dislocation storage.^[^ ^35^ ^]^ When the sintering temperature was lower than recrystallization temperature, extensive local plastic deformations of ZrB_2_ grains occurred and resulted in further highly localized shear, rotation, and the formation of small‐angle grain sub‐boundaries.

These morphologies clearly revealed the forming route of subgrains, and demonstrated that the mechanism of lattice distortion and dislocation slip could promote the formation of subgrains and plastic deformation of grains. The transmission electron microscope (TEM) results proved that the densities of dislocations and subgrains increased largely when the sintering temperature increased from 1350 to 1450 °C. The unique microstructure seems more favorable to enhance the properties and indicates that the optimum sintering temperature of ZrB_2_ under 15 GPa before recrystallization was 1450 °C.^[^ ^36^ ^]^

**Figure 3:**
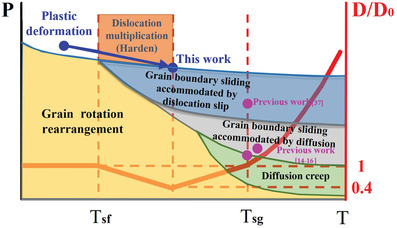
The summarized diagram of various densification mechanisms with the sintering temperature and applied pressure between particles for ceramics materials. The grain growth behavior in the present work is also included.

Based on the solid‐state sintering theory of ceramics and the previous studies, we summarized and established a densification mechanism diagram for sintering temperature and the real pressure between particles (**Figure** **3**). In our previous work, micro‐sized B_4_C and TaC, submicro‐sized Al_2_O_3_, and nano‐sized ZrO_2_ ceramics with limited grain growth have been fully densified under 80 MPa, 250 MPa, 200 MPa, and 1.5 GPa, respectively, by plastic deformation as the dominant densification mechanism.^[^ ^14^, ^15^, ^16^, ^37^ ^]^ The pressure‐assisted grain‐boundary diffusion or Coble creep help to eliminate the residue low porosity in the final sintering stage.^[^ ^14^, ^15^ ^]^ In this study, a developed densification mechanism of dislocation multiplication for ceramics was first observed. Through the formation of deformed stacking faults, substructures and high dislocation density under ultra‐high pressure, the ceramics could realize dense packing and densification by plastic deformation of grains. This mechanism could reduce the grain size and generate work‐hardening effect during sintering, which may develop the conventional sintering mechanism for ceramics. What's more, the effect of temperature on grain size under ultra‐high pressure was also slightly different from traditional sintering mechanisms. The occurrence of plastic deformation between particles contributed to densification without grain growth at low temperature. When the temperature increases to the starting temperature of grain refining (named as *T*_sf_) but lower than the critical temperature of grain recrystallization (named as *T*_sg_), there is still no much atom diffusion driven by thermal energy. The dislocation multiplication caused by plastic deformation resulted in the occurrence of substructures thus reduce the grain size. With the elevated temperature, the rapid grain growth induced by grain boundary migration was activated by heating when the temperature was above *T*_sg_.

### 2.3 Enhanced Mechanical Properties

**Table** **1** depicts the grain size, mechanical properties, and dislocation density of as‐preserved ZrB_2_ under different sintering and heat treatment conditions in the present work comparing with the available literatures. The hardness of ZrB_2_ ceramics sintered at 400 °C under 15 GPa was as high as 13.5 GPa, which was quite closed to that of ZrB_2_ prepared by hot press sintering in literatures.^[^ ^8^ ^]^ According to the classical sintering theory, the densification caused by atomic diffusion mainly relies on temperature and self‐diffusion coefficient. However, when the temperature is only 400 °C, there is very limited atomic diffusion driven by thermal energy, so the results confirmed that the densification mechanism was almost totally dependent on plastic deformation.

**Table 1:** The hardness, fracture toughness, grain size, and dislocation density of ZrB_2_ ceramics prepared under different conditions

Compositions	Sintering conditions	Heat treatment	Hardness [GPa]	Fracture toughness [MPa m^0.5^]	Grain size [µm]	Dislocation density [m^−2^]	Remark
ZrB_2_	2100°C‐35 MPa	–	16.02	2.98	6	–	^[^ ^8^ ^]^
ZrB_2_	2200 °C‐50 MPa	–	14.4	2.52	8	–	^[^ ^9^ ^]^
ZrB_2_	400 °C‐15 GPa	–	13.5±0.7	–	1.6 ± 0.10	–	Present work
ZrB_2_	1350 °C‐15 GPa	–	24.1 ± 1.0	3.05 ± 0.18	1.1 ± 0.10	1.38 × 10^15^	Present work
ZrB_2_	1450 °C‐15 GPa	–	25.4 ± 1.2	3.25 ± 0.15	0.7 ± 0.09	1.7 × 10^15^	Present work
ZrB_2_	1600 °C‐15 GPa	–	22.9 ± 0.7	2.62 ± 0.13	1.6 ± 0.12	5.6 × 10^14^	Present work
ZrB_2_	1700 °C‐15 GPa	–	20.5 ± 0.9	2.24 ± 0.15	2.15 ± 0.20	9.25 × 10^13^	Present work
ZrB_2_	2000 °C‐200 MPa	–	17.4 ± 0.8	1.92 ± 0.14	19 ± 1.33	2.78 × 10^12^	Present work
ZrB_2_	1450 °C‐15 GPa	1750 °C‐90 min	19.7 ± 0.4	2.15 ± 0.13	2.8 ± 0.30	4.07 × 10^13^	Present work

The sample sintered under 15 GPa at 1450 °C showed the optimized mechanical properties, which possesses the hardness of 25.4 GPa, fracture toughness of 3.25 MPa·m^0.5^, with the finest grain size of 0.7 µm and highest dislocation density of 1.7 × 10^15^ m^−2^. Compared with the SPS‐ed ZrB_2_, the grain size of UHPS‐ed ZrB_2_ reduced above 96%, while the hardness and fracture toughness increased about 46% and 69%, respectively. In addition, the mechanical properties of samples in the present work were significantly high than that in previous studies in Table 1.^[^ ^8^, ^9^, ^10^ ^]^ The hardness and toughness could be promoted simultaneously by adjusting the sintering parameters: dislocation density and sub‐structures. Hu modified the hardness and toughness of steel at the same time by refining the dislocation substructures and defined this phenomenon as dislocation engineering.^[^ ^38^, ^39^ ^]^ The results in the present work suggested that similar strategy could be utilized to improve the performance of ceramics.

In the following discussion, the ZrB_2_ optimally sintered at 1450 °C under 15 GPa was used to represent the ultra‐high pressure sintered sample, which was marked as UHPS‐ZrB_2_, while the control sample sintered at 2000 °C with 200 MPa by SPS was marked as SPS‐ZrB_2_.

### 2.4 The Effect of Dislocation Regulation on Mechanical Properties

**Figure 4:**
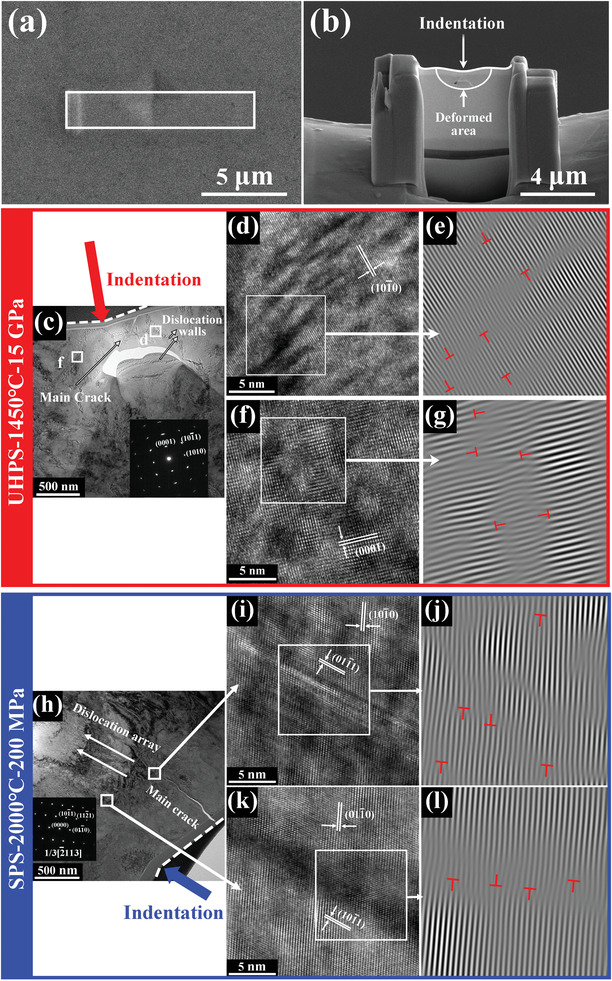
a) The microstructure of the indentation impression. The white rectangle area reflects sampling location corresponding to the indentation. b) SEM image indicating the cross‐sectional morphology of the ZrB_2_ TEM foil taken after a 250 mN micro‐Vickers hardness test. Upper arrow shows the indented direction and semi‐elliptical area reflects the deformation area. The morphology of UHPS‐ZrB_2_ after indentation‐induced deformation: c) The bright field with SAED patterns inserting; d,f) HRTEM micrographs of dislocations corresponding to the position shown in (c); e,g) IFFT images of the boxed regions showing the obvious dislocations among the grains in (d,f), respectively. The morphology of SPS‐ZrB_2_ after indentation‐induced deformation: h) The bright field image with SAED patterns inserting; i,k) HRTEM micrographs of dislocations corresponding to the position shown in (h); j,l) IFFT images of the selected regions showing the obvious dislocations in (i) and (k), respectively.

To illustrate the effect of dislocation regulation on the mechanical properties, the focused ion beam milling (FIB) technique was used to slice the residual indentation of UHPS‐ZrB_2_ after the microhardness test with the applied load of 250 mN (**Figure** **4**). The selected machining region corresponds to the indentation position, which could reflect the deformation‐resisted properties and mechanism.^[^ ^40^ ^]^

Figure 4c–g shows the TEM characterization of UHPS‐ZrB_2_ at the deformed area after indentation. The arrows in bright field show the specific dislocation walls (Figure 4c). The submicro‐sized pores in the deformation area were attributed to the intrinsic brittleness of the ceramic in the failure process. The grains underwent severe plastic deformation, as indicated by the SAED patterns in Figure 4c.

Dislocation walls in different directions were the main defects generated during indention‐induced deformation (Figure 4d–g) and could inhibit further plastic deformation and brittle fracture caused by crack propagation. Varieties directions of dislocation walls presented in the ZrB_2_ foils suggested that the plastic deformation densification mechanism resulted in the interaction among dislocations and enhanced the work‐hardening effect. The small‐angle grain boundaries of ZrB_2_ ceramics form a lot of interfaces thus increasing the energy required for crack propagation and improving mechanical properties of the material.^[^ ^40^ ^]^

Dislocation arrays in the control sample of SPS‐ZrB_2_ after indentation‐induced deformation (Figure 4h–l) suggested that plastic deformation and brittle failure took place during indentation. The propagation direction of the main crack was parallel to the loading direction of indentation. The IFFT patterns taken from the main crack tip (Figure 4i,j) and the deformation area (Figure 4k,l) respectively displayed a series of edge dislocations and stacking faults, confirming that the dislocations only moved along the (10$\bar{1}$1) plane. Shear‐induced dislocation movement and the crack propagation preferentially took place along the (10$\bar{1}$1) plane parallels to the loading direction of the indentation.

When the UHPS‐ZrB_2_ underwent indention‐induced damage, due to the high‐density disordered dislocation, the stress‐induced dislocation motion was impeded and the slip directions were changed. The IFFT patterns show that a series of dislocation arrays moved along various crystal planes. The morphology indicates that for UHPS‐ZrB_2_, the slip systems with various moving directions occurred in the deformation area and at the crack tip, whereas only one slip system was observed in SPS‐ZrB_2_ with the same direction of indentation. It appears that the dislocation density in ZrB_2_ ceramics affected the hardness and fracture toughness by controlling the movement of dislocation arrays generated by plastic deformation.^[^ ^38^ ^]^ In this section, the microstructure observation directly proved that the disordered dislocations induced by UHPS hindered the crack extension, thus improving the mechanical properties.

### 2.5 The Effects of Annealing on Mechanical Properties

**Figure 5:**
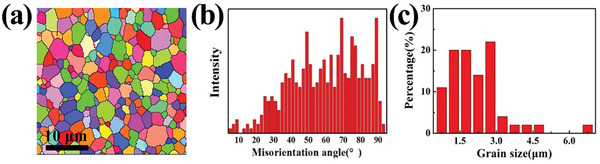
The EBSD analysis of ZrB_2_ ceramics sintered at 1450 °C under 15 GPa annealed at 1750 °C for 90 min. a) EBSD image. b) Misorientation angle distributions and c) grain size distribution.

To illustrate the influence of annealing on the mechanical properties, the sample sintered under 15 GPa at 1450 °C was subsequently heat treated at 1750 °C for 90 min.^[^ ^41^ ^]^ The microstructure after heat treatment is shown in **Figure** **5**. The dislocation density decreased 2 orders of magnitudes to 4.07 × 10^13^ m^−2^. The hardness and fracture toughness reduced 22% (to 19.7 GPa) and 33% (to 2.15 MPa·m^0.5^), respectively. In addition, a large amount of small‐angle grain boundaries, lattice distortion, and substructures in the UHPS‐ZrB_2_ sample disappeared. The result indicates that subgrains were transformed into new grains because of activated grain boundary diffusion. The decrease of hardness and toughness after heat treatment decreased due to the increase in grain size and the reduction of dislocation density at high temperature.

An interesting grain growth behavior was found during the heat treatment process. In pure ZrB_2_ polycrystalline ceramics sintered at 1750 °C, grains did not clearly grow even under the additional driving force from the applied stress.^[^ ^11^ ^]^ But here the average grain size increased 4 times from 0.7 to 2.8 µm. It was proposed that the grain growth could only be driven from the energy storage of plastic deformation during UHPS process. The microstructure evolution also confirmed that the grain growth was induced by static recrystallization.^[^ ^42^ ^]^ The increase of grain size could lead to decrease of hardness. The relationship among the grain mobility energy during the static recrystallization, the grain size, and the annealing time are shown in Equation (4).^[^ ^41^ ^]^

(4) $$G_{t}^{2}-\;G_{0}^{2}=\frac{\gamma_{\mathrm{GB}}M_{\mathrm{GB}}}{4}\;t$$

where *G*_0_ and *G_t_* are respectively the grain sizes of UHPS‐ZrB_2_ before and after annealing, respectively; *γ*_GB_ is the grain boundary energy; *M*_GB_ is the grain boundary mobility; *t* is the annealing time. The average grain boundary mobility rate caused by the storage energy of plastic deformation during heat treatment at 1750 °C was calculated as 3.4 × 10^−16^ m^2^ s^−1^, suggesting that the grain growth was induced by plastic deformation for ceramics.

The high dislocation density and refined grains were induced by ultra‐high pressure. The unique dislocations and internal prestress made a special contribution to hardness and fracture toughness. However, the grains became equiaxial and the grain size increased due to the occurrence of grain boundary mobility and recrystallization after annealing. In this way, the mechanical properties were weakened. Therefore, grain refinement with prestress during ultra‐high pressing show positive effects on hardness and fracture toughness of ZrB_2_ ceramics.

### 2.6 Relationship among Hardness, Grain Size, and Dislocation Density of ZrB2 Fabricated by Ultra‐High Pressure Sintering

**Figure 6:**
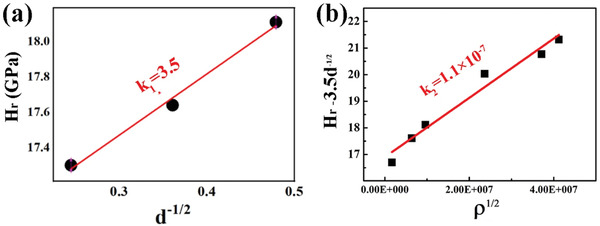
a) *H* as a function of average grain size based on the previous studies.^[^ ^8^, ^9^, ^10^ ^]^ b) *H*‐3.5*d* ^−1/2^ as a function of dislocation density for polycrystalline ZrB_2_ ceramics based on our experimental data in the present work.

With the decrease in grain size and the increase in dislocation density, the hardness of polycrystalline ZrB_2_ ceramics monotonically increased.^[^ ^42^, ^43^, ^44^ ^]^ The Hall–Petch effect provides guidance for a widely used approach to produce ceramics with fine microstructure. It confirms that the strength and hardness enhancement is inversely proportional the square root of average crystallite grain size. It is reported that, for polycrystalline ceramics, the hardness and grain size agree with Hall–Petch unless the grain size is beyond 600 µm.^[^ ^44^ ^]^ Additionally, according to the Taylor hardening effect, the macroscopic hardness is proportional to the square root of dislocation density. The dislocations are assumed as barriers to slip, thus leading to strain hardening and strengthening.^[^ ^45^ ^]^ Based on the combination of Hall–Petch and Taylor effect, the relationship between hardness enhancement, grain size, and dislocation multiplication of ceramics in the study can be expressed as followed:

(5) $$\;H_{r}=H_{0}\;+k_{1}d^{-\frac{1}{2}}+k_{2}\rho_{d}^{\frac{1}{2}}$$

where *H*_r_ is the hardness; *H*_0_ is the intrinsic hardness; *d* is the grain size; *ρ*_d_ is the dislocation density; *k*_1_ *and k*_2_ are respectively the slopes of Hall–Petch and Taylor hardening effects. To determine the parameter *k*_1_ for ZrB_2_, we summarized the hardness data of a series of ZrB_2_ ceramics with various grain sizes and relative densities. Only the studies based on pure ZrB_2_ powder were selected for the analysis.^[^ ^8^, ^9^, ^10^ ^]^ In addition, because the dislocation density of SPS‐ed ZrB_2_ was quite small even under 200 MPa, the effect of dislocation density on the ZrB_2_ ceramics prepared previously under the applied load ranging from 25 to 50 MPa could be ignored and their hardness was completely determined by grain size. However, it is difficult to obtain the fully dense single‐phase ZrB_2_ ceramics by conventional method such as pressureless sintering, SPS, and HP. In order to eliminate the influence on hardness by porosity, the true value of hardness (*H*_r_) was calibrated based on the following minimum solid area models^[^ ^46^ ^]^:

(6) $$\;H_{r}=\;H_{e}\exp\left[-k_{3}\left(1-\rho \right)\right]$$

where *H*_e_ is the measured hardness; *ρ* is the relative density; *k*_3_ is a constant and set as 7.^[^ ^46^ ^]^ **Figure** **6**a shows the inversely proportional relationship between hardness and the square root of grain diameter determined in previous reports.^[^ ^8^, ^9^, ^10^ ^]^ The value of *k*_1_ is calculated as 3.5.

The results based on the experimental data in the present work confirmed the relationship between hardness and dislocation density. The *k*_2_ constant was calculated as 1.1 × 10^−7^ from the slope between *H*‐3.5*d* ^−1/2^ and *ρ*_d_^1/2^. According to Taylor hardening law shown in Equation (5), the hardness induced only by dislocation strengthening was estimated to be about 4.8 GPa.

Based on the above analysis, the relationship between hardness, grain size, and dislocation density of ZrB_2_ ceramics fabricated by UHPS follows Equation (7).

(7) $$H\;=H_{0}\;+3.5d^{-\frac{1}{2}}+1.1\times 10^{-7}\rho_{d}^{-\frac{1}{2}}$$

### 2.7 The Promotion of Oxidation Resistance

**Figure 7:**
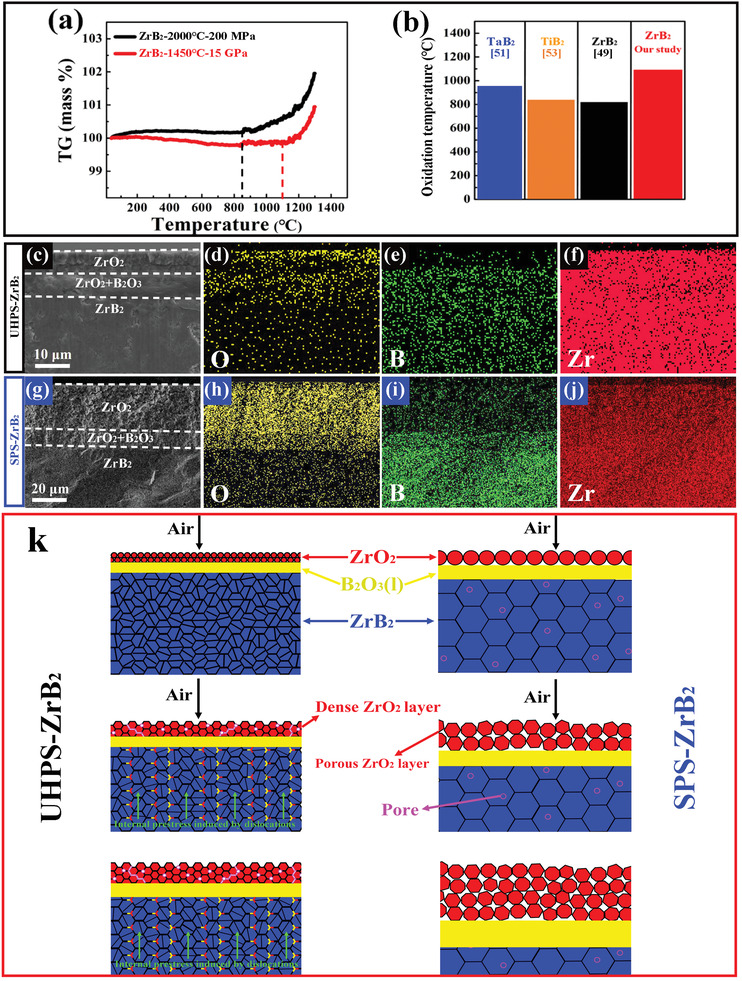
Thermal analysis on UHPS‐ZrB_2_ and SPS‐ZrB_2_ in air atmosphere: a) TG curves, b) comparison of the onset oxidation temperatures of UHPS‐ZrB_2_ with SPS‐ZrB_2_ and other reported ultra‐high temperature ceramics including TaB_2_, TiB_2_, and ZrB_2_. Cross section morphologies and EDS mapping images of the as‐sintered ZrB_2_ ceramics after oxidation in air for 0.5 h at: c–f) 1100 °C for UHPS‐ZrB_2_, g–j) 850 °C for SPS‐ZrB_2_. k) The oxidation diagram under different temperatures of the ZrB_2_ sintered by different method. The left half represents the oxidation process at 1100 °C for 1450 °C‐15 GPa UHPS‐ed ZrB_2_, while right half shows the process at 850 °C for 2000 °C‐200 MPa SPS‐ed ZrB_2_.

The oxidation resistance of ZrB_2_ ceramics sintered by UHPS of 1450 °C‐15 GPa, and SPS of 2000 °C‐200 MPa was respectively characterized by thermal analysis in air with a heating rate of 5 °C min^−1^ (**Figure** **7**). The increase in the mass could be attributed to the reaction in Equation (8), which could also determine the oxidation process of ZrB_2_.^[^ ^47^ ^]^ The onset temperature of oxidation for UHPS‐ZrB_2_ was as high as 1100 °C, while it was only 850 °C for SPS‐ZrB_2_.

(8) $$\mathrm{Zr}B_{2}\left(s\right)+\frac{5}{2}O_{2}\left(g\right)\to \mathrm{Zr}O_{2}\left(s\right)+B_{2}O_{3}\left(l\right)$$

Further comparison is also included in Figure 7b with the as‐preserved ZrB_2_ ceramics in the present work and monolithic ultra‐high temperature ceramics prepared by other researchers. The UHPS‐ZrB_2_ started to show obvious oxidation at the temperature of 1100 °C, which was much higher than that of SPS‐ZrB_2_ (850 °C) and other reported ultra‐high temperature ceramics such as TaB_2_ (930 °C), ZrB_2_ (800 °C), and HfC (700–900 °C).^[^ ^48^, ^49^, ^50^, ^51^, ^52^, ^53^, ^54^ ^]^

According to the results of oxidation behavior analysis, UHPS‐ZrB_2_ and SPS‐ZrB_2_ were thermally annealed for 0.5 h respectively at 1100 and 850 °C in air atmosphere in a muffle furnace to compare the microstructure evolution and elemental distributions after oxidation.

The SEM images and EDS images of the cross sections of samples oxidized at 1100 °C for UHPS‐ZrB_2_ and 850 °C for SPS‐ZrB_2_, respectively, are shown in Figure 7c–j. The as‐preserved ZrB_2_ were oxidized in air at elevated temperatures. The formed oxide layer covered the surface of the sample. Elemental analysis indicates that the structures of oxide layers for both ZrB_2_ ceramics were composed of an outside ZrO_2_ layer without B_2_O_3_, a middle‐layer containing the mixture of ZrO_2_ and B_2_O_3_, and the inside ZrB_2_ with very limited oxidation. However, the thicknesses of oxide layers for the two ZrB_2_ ceramics were quite different.

The ZrO_2_ layer of UHPS‐ZrB_2_ oxidized at 1100 °C shows the thickness of 15 µm, whereas in the oxidized SPS‐ZrB_2_, it is as much as 40 µm even under much lower temperature of 850 °C. The results confirmed the much better oxidation‐resistant properties for ZrB_2_ ceramics prepared by UHPS technology.

The oxygen diffusion rate is considered as the most important factor to control the oxidation rate for ZrB_2_ ceramics.^[^ ^48^, ^49^ ^]^ Figure 7c shows that after oxidation at 1100 °C, the ZrO_2_ layer of oxidized UHPS‐ZrB_2_ is dense and fine. The morphology characterization and elemental mapping suggested that the nano‐sized substructures of UHPS‐ZrB_2_ were transformed into fine ZrO_2_ grains, and then sintered at relative low temperature. The densified ZrO_2_ could act as a protective layer to prevent further oxygen diffusion into the interior. In addition, the unique microstructure and internal stress of UHPS‐ZrB_2_ might play a positive role in preventing further oxidation. In contrast, as shown in Figure 7g, the ZrO_2_ layer of SPS‐ZrB_2_ contained lots of large pores, which could not hinder oxidation.

Figure 7k shows the proposed diagram of oxidation process for the as‐preserved ZrB_2_ at different temperatures. It suggests that the better oxidation resistance for UHPS‐ed ZrB_2_ than SPS‐ed ZrB_2_ could be attributed to three reasons. First, the grain size of UHPS‐ed ZrB_2_ was much smaller, leading to the thinner initial oxidation layer. The increasing heating temperature led to the densification of nano‐sized ZrO_2_, which prevented further oxidation. In comparation, the loose oxidation layer with large‐sized ZrO_2_ particles formed at the surface of SPS‐ed ZrB_2_ could not play a practical role for oxidation barrier. Second, the UHPS‐ed ZrB_2_ ceramics had a lot of dislocations and substructures, which may produce prestress to restrain further oxidation. The SPS‐ed ZrB_2_ without substructures could facilitate oxygen diffusion. Third, the oxide films were formed at most of the trigonal grain boundaries with higher chemical potential and internal stress to prevent oxygen from entering the inner grains. But for SPS‐ed ZrB_2_, the existence of isolated intracrystalline pores may even promote the oxidation by supplying diffusion channel for oxygen.

The high dislocation density, substructures, and prestress in as‐preserved ZrB_2_ indicated that UHPS was an effective route for simultaneous enhancement of the mechanical and oxidation‐resistant properties of ceramic materials. Based on the intrinsic characteristics and the unique microstructure of ZrB_2_ in the present study, it may be suitable for other various applications besides at high temperature, such as electrodes, cutting tools, electrical devices, and nuclear component, which need to be studied in the future.^[^ ^55^, ^56^ ^]^

## 3 Conclusion

In summary, fully‐dense and finer‐grained ultra‐high temperature ZrB_2_ ceramics were successfully fabricated under ultra‐high pressure at low temperature. The dominant densification mechanism was proposed and proved as the plastic deformation‐induced dislocation multiplication and grain boundary sliding. The proportion of dislocation cells, substructures, and small‐angle grain boundaries gradually increased when the sintering temperature reached the critical temperature of 1450 °C. ZrB_2_ sintered under 15 GPa at the optimized temperature of 1450 °C holding 10 min showed full density, fine microstructures, high dislocation density, and excellent mechanical properties. The grain size of as‐sintered ZrB_2_ ceramics decreased ≈56% compared with the raw powder. The microstructure observation after indentation and properties characterization after heat treatment proved that the high dislocation density, finer grain size, and high prestress induced by UHPS contributed the enhancement of mechanical properties. In addition, the UHPS‐ed ZrB_2_ exhibited excellent oxidation resistance. The nano‐sized ZrB_2_ subgrains at the surface were oxidized into fine ZrO_2_ and then densified at relative low temperature, which could prevent further oxidation.

## 4 Experimental Section

### Sintering Process

The experimental synthesized ZrB_2_ (mean particle size: 1.6 µm, oxygen: 0.96%) was chosen as the raw materials. The procedure was introduced in the authors’ previous work.^[^ ^21^ ^]^ UHPS experiments were performed with a Type‐Kawai large‐volume multi‐anvil system (LPRU1000, Max Voggenreiter, Germany). The assembly was composed of spinel octahedron, Re heater, LaCrO_3_ thermal insulator, and Al_2_O_3_ sleeve. The temperature under high pressure was monitored in situ with a Type C W‐Re thermocouple. ZrB_2_ powder was compressed to 15 GPa and heated in the temperature range of 400–1700 °C for 10 min and the heating rate was 100 °C min^−1^. In order to clarify the contribution of pressure on the densification process, a sample was sintered at 400 °C under 15 GPa.

ZrB_2_ ceramics fabricated by SPS were used as the control samples. The powder was loaded in a carbon fiber reinforced carbon composite (C_f_/C) die with an inner diameter of 10 mm then sintered for 5 min with a heating rate of 200 °C min^−1^ at 1800 °C under 50 MPa, and 2000 °C under 200 MPa by using a SPS system (ED‐PAS‐111, ELENIX, Japan).

### Characterization

The morphologies of fracture surface of samples were observed by a field emission scanning electron microscope (Quanta FEG 250, FEI, USA). All the as‐sintered specimens were grinded and polished to 0.25 µm finish with diamond paste. The microstructures of the polished surfaces were examined with an EBSD (Symmetry EBSD, Oxford Instruments, UK). EBSD was used to analyze the local crystallite lattice orientations and lattice curvature data to estimate the dislocation density. A TEM (Talos F200S, FEI, USA) was used to investigate the detailed microstructures and grain boundaries of as‐prepared ZrB_2_ samples. The ZrB_2_ foils used for TEM test were prepared by an ion‐beam thinning equipment (EM RES102, Leica, German).

The Vickers hardness (*H*_v_) and fracture toughness (*K*_IC_) were measured with a micro‐hardness tester (Tukon 2100, Wilson‐Wolpert, USA) under the load of 1 kg with a dwelling time of 15 s, according to the following equations:^[^ ^20^ ^]^

(9) $$H_{v}\;=\;1854.4\frac{P}{L^{2}}$$

(10) $$K_{\mathrm{IC}}=\;0.16H_{v}\sqrt{L}{\left(\frac{c}{L}\right)}^{-\frac{3}{2}}$$

where *P* is the applied load; *L* is the arithmetic mean of the lengths of the two diagonals of the Vickers indentation; *c* is the average value of the radial cracks measured from the indent center.

For further analysis, the effect of microstructures including dislocation density and directions on mechanical properties, the micro‐Vickers hardness device, a constant load of 250 mN was applied on a polished ZrB_2_ ceramic for 15 s to form the deformation fracture area. The cross‐sectional ZrB_2_ TEM foils of the deformed areas were sliced with the FIB apparatus (Helios NanoLab G3 UC, USA).

To study the effect of annealing on the properties, the UHPS‐ed ZrB_2_ was heated at 1750 °C for 90 min through Ar atmosphere using a high temperature furnace (Thermal Tech Co. Ltd, Santa Rosa, USA).

The oxidation resistance properties of ZrB_2_ were studied with the thermogravimetric analysis‐differential scanning calorimeter (NETZSH STA449F3, Germany) in air atmosphere via the simultaneous thermal analysis technique and the temperature was heated to 1300 °C with a heating rate of 5 °C min^−1^. The thermogravimetric (TG) curves were recorded and used to present oxidation behavior.

### Statistical Analysis

The average grain size was determined by measuring 100 grains via nano measure software. The reported values of Vickers hardness and fracture toughness were the average of at least six measurements. The EBSD and TEM results were analyzed via Channel 5 and Gatan Digital Micrograph software, respectively.

## Conflict of Interest

The authors declare no conflict of interest.

## Data Availability

Research data are not shared.
